# Behaviour sequelae following acute Kawasaki disease

**DOI:** 10.1186/1471-2431-5-14

**Published:** 2005-05-25

**Authors:** Daniel Carlton-Conway, Raju Ahluwalia, Lucy Henry, Colin Michie, Louise Wood, Robert Tulloh

**Affiliations:** 1 Department of Paediatric Cardiology, Royal Hospital for Children, Upper Maudlin Street, Bristol, BS2 8BJ, UK; 2 Department of Paediatric Cardiology, Guy's and St Thomas' Hospital Trust, Guy's Hospital, St Thomas' Street, London, SE1 9RT, UK; 3 Department of Paediatrics, Ealing Hospital, Uxbridge Road, Southall, Middlesex, UB1 3EW, UK; 4 Institute of Psychiatry, de Crespigny Park, London, SE5 8AF, UK

**Keywords:** Kawasaki Disease, Cerebral Vasculitis, Psychological Difficulties, Long Term Management

## Abstract

**Background:**

Kawasaki disease is a systemic vasculitis and may affect cerebral function acutely. The aim of the present study was to measure a number of behaviour and social parameters within a cohort of Kawasaki disease patients.

**Methods:**

Parents of children with past diagnosis of Kawasaki disease were recruited to complete several behaviour screening questionnaires. Sixty five sets of questionnaires relating to the patient cohort received were eligible for inclusion. Two control groups were used, a hospital (HC) control and a sibling control (SC) group.

**Results:**

40% of the Kawasaki disease group showed elevated internalising scores in the clinical or borderline-clinical range. This compared with 18% of hospital controls and 13% of sibling controls. Additionally, the Kawasaki disease (KD) group were shown to be experiencing greater overall total difficulties when compared with the controls (KD 13.7, HC 8.6, SC 8.9). The KD group attained higher behavioural scores within the internalising sub-categories of somatic problems (KD 61, HC 57, SC 54) and withdrawn traits (KD 56, HC 53, SC 51). The KD group were also shown to be suffering more thought problems (KD 57, HC 53, SC 50) compared with the controls. Further difficulties relating to conduct (KD 3.3, HC 1.4) and social interactions (KD 6.7, HC 8.3) are also highlighted for the KD group compared with hospital controls. Positron emission tomograms were performed on nine patients to investigate severe behavioural problems. Three showed minor changes, possibly a resolving cerebral vasculopathy.

**Conclusion:**

Kawasaki disease can be associated with significant behavioural sequelae. This is an important consideration in the long-term follow up and referral to a clinical psychologist may be necessary in selected patients.

## Background

In 1967, Tomisaku Kawasaki first described an illness in children called 'acute febrile mucocutaneous lymph node syndrome' [1]. It has now become more commonly known as Kawasaki disease. The diagnostic criteria according to the Centres for Disease Control (Atlanta) are laid out below. Fever of 5 days plus 4 out of the 5 remaining criteria [2], or fever plus coronary artery aneurysms at echocardiography and 3 additional criteria are generally required for a diagnosis of Kawasaki disease to be made [3] (see table 1).

**Table 1:** Criteria necessary to confirm diagnosis of Kawasaki disease [2]

**Criteria**	**Description**
Fever	5 days or more
***AND***	
1. Conjunctivitis	Bilateral
2. Mucous membrane changes of oropharynx	Red cracked lips; strawberry tongue; or diffuse erythema
3. Changes of peripheral extremities	Initially: erythema and oedema of palms and soles Convalescent stage: desquamation
4. Rash	Polymorphous, non vesicular
5. Lymphadenopathy	Cervical

Cardiac complications can occur which include coronary artery aneurysms, which have been documented as developing during the illness in 25% of patients treated with aspirin alone (determined at echocardiography) [3,4]. Treatment with intravenous gammaglobulin and aspirin has been shown to reduce this incidence considerably [5,6].

Pathologically, Kawasaki disease is a multisystem vasculitis affecting small and medium sized arteries. Inflammation is progressive, initially mild affecting only the subendothelium and progressing to a severe panarteritis of the coronary arteries. Injured arteries are weakened and thus aneurysms may form as a result [3].

Neurological and psychological complications associated with Kawasaki disease have also been noted, although reports in this area are limited. Neurological complications include aseptic meningitis occurring in 26–50% of cases as well as facial nerve palsy, sensorineural hearing loss, hemiplegia, cerebral infarction and severe lethargy, which have all been reported in various case studies [3-6]. Amano and Hazama have examined histological specimens from 30 Kawasaki disease patients and found evidence of the occurrence of endoarteritis and periarteritis within the brain [3] and it is likely that many of the CNS complications are secondary to such a process of cerebral vasculitis [8].

Extreme irritability is a well documented observation seen in children during the acute phase of the illness, which generally resolves after treatment. However, a recent study performed by King and workers have reported that behavioural problems may develop or persist once the acute phase of the illness has passed and then continue for months or years after the initial illness [7]. The study consisted of 32 patients with Kawasaki disease in the USA and used siblings as controls. They demonstrated that post acute Kawasaki disease patients had a significantly greater tendency to internalising and attentional behavioural problems, although no firm aetiology was uncovered [7]. However, this study was somewhat limited as it was carried out on a relatively small cohort and did not include a control group of patients that had been hospitalised for a similar time period.

It is these behavioural sequelae that we are interested in as we have documented many cases in clinic of reported behavioural problems persisting for months and years after the initial episode of Kawasaki disease.

We initially set out to screen for potential behavioural problems within a British patient cohort. 123 children with a past diagnosis of Kawasaki disease reviewed in a long term Kawasaki disease follow up clinic were included. Parents were asked at time of their visit to answer a series of questions relating to behaviour and certain aspects of their child's disease. Additionally, 142 questionnaires used by a national Kawasaki disease parent support group were reviewed retrospectively. All the data was collected between 1996–1999, with 265 children being reviewed in total. The questions in clinic and within the help group questionnaire were derived from Rutter's B questionnaire, with the aim to identify significant changes in physical and psychological health as perceived by the child's carer [3]. The results demonstrated that 90 of the children (34%) suffered from behavioural changes, lasting for more than one year following their recovery from the acute stages of Kawasaki disease. These behavioural problems were significant enough to lead to consultation with an educational psychologist, clinical psychologist or a GP. The behavioural problems recognised in the data included hyperactivity, decreased concentration, increased aggression and emotional lability.

Based on these initial results, we therefore set out to investigate these behavioural difficulties in a more detailed and quantitative fashion within a British Kawasaki disease patient cohort.

There is nothing in the current management protocol that is aimed at addressing behavioural difficulties, nor in the advice given to parents. Finding a significant correlation between Kawasaki disease and behavioural sequelae would provide the basis for an important added dimension in the long-term management of the disease. We would hope to provide a greater insight and awareness into behavioural sequelae, which would lead to earlier referrals to a child psychologist where necessary, more specific advice to the parents on how best to manage their child as well as providing reassurance that the child's behavioural difficulties may be within the normal sequelae of Kawasaki disease.

## Methods

A retrospective cohort study was selected as the most appropriate method in view of the uncommon frequency of Kawasaki disease within the UK population. Parents of children who had been seen in clinic between 1995 – 2001 were contacted by telephone and informed verbal consent gained. When parents could not be contacted by phone, a detailed covering letter explained the nature of the research was sent out and parents were invited to respond. The study was approved by the Local Ethics Committee.

### The Kawasaki disease patient group (KD group)

Inclusion criteria for this stage of the research were: (a) all patients aged under 18 years at time of diagnosis and at the time of the study were between 3 to 18 years old; (b) A diagnosis of KD formally having been reached according to the criteria in table 1; (c) sufficient level of English spoken by parent/carer to understand questionnaires; and (d) no history of CNS dysfunction unrelated to KD, or any other history of severe illness.

112 sets of questionnaires were sent out to parents. 77 questionnaires were received and 65 were suitable for inclusion according to our predetermined criteria.

### Controls

Two control groups with different characteristics were used. The first consisted of previous hospital inpatients who had stayed in hospital for a short period and undergone a cardiac catheterisation, with follow-up at regular intervals. This group was chosen in order to control for the effects of a hospital stay on child behaviour. Patients who had subsequently progressed to surgery, or suffered from an unrelated CNS disorder or other chronic illness were not deemed suitable for inclusion. These controls were matched to the KD group, as best as possible, according to age and sex. 47 sets of questionnaires were sent out and 23 were returned completed.

The second control group consisted of siblings of the KD group matched for age and sex, as best as possible. This control group was selected in order to control for the influence of genetics and environment on child behaviour. 31 sets of questionnaires were sent out and 17 were returned.

Details of the groups are included in Table 1.

### Data collection instruments

Mothers of patients were sent out a series of psychological screening questionnaires for behavioural difficulties, which they were invited to complete. The questionnaire set comprised: the Child Behaviour Check List – CBCL/4–18 (*Achenbach 1991*) [13]; Strengths & Difficulties Questionnaire *(Goodman 1997) *[14], and the Parenting Stress Index – PSI, (*Abidin 1983*) [15]. A general information sheet was also devised for inclusion with the questionnaires.

The Child Behaviour Check List (CBCL) tests the parental reports of a child's competencies and quantifies behavioural and emotional problems along predetermined categories. It has a one week test-retest reliability of r = 0.93 [13]. The CBCL was used to obtain standardised reports from parents on behavioural and emotional problems for their children. The CBCL contains 120 problems items and measures in two broad categories: Internalising problems (consisting of the scales: anxious/depressed, somatic complaints and withdrawn behaviour) and Externalising complaints (aggressive, and delinquent behaviour). Additional scales of social problems, thought problems and attention problems are also included.

The parenting stress index (PSI) short form is designed to measure stresses occurring within the parent-child relationship and provides insight as to whether any difficulties originate from the parent, the child or their interaction. It uses the following categories; Total stress, Parental distress, Parent child dysfunctional interaction, and Difficult child. Also, scores for defensive responding were recorded.

The Strengths and Difficulties Questionnaire (SDQ), looks at behavioural, emotional and/or relationship difficulties in children as well as a positive category that rates social strengths and interactions. The SDQ provides scores across 5 subscales; conduct, hyperactivity, emotional, peer, and prosocial problems. It also generates a total difficulty score.

### Statistical analysis

One-way analyses of variance (ANOVA) were conducted on each measure, with Bonferoni post hoc testing to examine the differences between the groups. Fisher's Exact Test was also employed to test the strength of a trend within the groups. Means for each measure and standard errors are given in Table 2.

**Table 2:** Comparison between patient cohort and controls

	**KD patient Group**	**Hospital Controls**	**Sibling Controls**
Number in group	65	23	17
Male : Female	1.6:1	0.9:1	0.9:1
Age on completion (mean, range)	7.38 (3–18)	8.95 (3–17)	8.68 (5–14)
Patient age at presentation with Kawasaki disease (median, range)	3.60 (0–14)	N/A	N/A
Number with coronary artery dilatations or aneurysms secondary to Kawasaki disease	13 (20%)	N/A	N/A
Reported difficulties (%)	56.3%	35.3%	46.2%

## Results

When asked by a single question, 56% of the Kawasaki disease patient cohort were reported to be experiencing minor to severe difficulties: emotionally, behaviourally or socially. This compared with 35% of hospital controls and 46% of sibling controls. Looking at the scores on the Child Behaviour Check List (CBCL), the KD group consistently achieved higher mean behavioural scores than the two control groups within all categories. (Table 2).

Significantly higher behavioural scores were attained by the KD group within the withdrawn (P = 0.045) and somatic (P = 0.030) categories, when compared with the sibling controls (Table 3). This suggests a greater tendency towards internalising behaviour within the KD group. Indeed, 40% of the KD group fell within the clinical or borderline range for internalising problems, compared with 18.2% of the hospital controls and 12.5% of the sibling controls (FE = 0.040). No significant differences between the groups were found for externalising problems. The KD group was found to have significantly higher behavioural scores on the thought problem category of the CBCL compared with the sibling control group (P = 0.006). Thought problems reported included obsessions, compulsions as well as strange behaviour. Results from the social and schooling categories of the CBCL did not suggest any significant differences between the three groups.

**Table 3:** Selected results, including all that were significant, from the scores to the questionnaires. FE = Fisher's exact test; SEM = Standard Error of Mean; † indicates P values less than 0.05 * indicates which control groups differed significantly (P < 0.05) from the KD group (see text for p values)

**Category (mean, SEM)**	**KD Group**	**Hospital Controls**	**Sibling controls**	**Significance **(ANOVA unless indicated)
**CBCL/4–18**				
Internalising score in clinical or borderline range	40.0%	18.2%	12.5%	**0.04 † (FE)**
Externalising score in clinical or borderline range	26.2%	9.1%	25%	**0.27 (FE)**
Thought Problems	57.0 (1.06)	52.8 (1.19)	50.2 (1.74) *	**0.03 †**
Withdrawn	55.9 (0.96)	52.8 (1.19)	51.2 (0.82) *	**0.02 †**
Somatic	61.0 (1.27)	57.3 (1.63)	54.1 (1.81) *	**0.02 †**
Sex Problems	54.0 (1.10)	52.9 (1.57)	47.3 (1.28) *	**0.01 †**
Aggressive	56.5 (1.19)	52.0 (0.71)	52.6 (2.07)	0.05
Internal T score	53.4 (2.0)	48.5 (2.38)	44.1 (2.81)	0.05
Total T score	53.4 (1.73)	48.4 (2.45)	45.6 (3.36)	0.07
**Strengths + Difficulties**				
Prosocial	6.7 (0.31)	8.3 (0.48) *	7.4 (0.63)	**0.04 †**
Conduct	3.3 (0.38)	1.4 (0.34) *	2.8 (0.73)	**0.04 † **
Total Difficulties	13.7 (1.08)	8.6 (1.44) *	8.9 (1.93)	**0.02 †**
**Parenting Stress Index**				
Difficult child (raw)	30.3 (1.52)	24.2 (1.47)	26.5 (2.80)	0.07

Scores on the Strengths and Difficulties Questionnaire showed the KD group achieving higher mean scores than both control groups within the four negative categories of conduct, peer, emotional and hyperactivity. Along the positive prosocial category (rating social strengths and interactions) the KD group achieved a lower mean score than both the control groups. The KD group were significantly less helpful and considerate (prosocial category) than the hospital control group (P = 0.045). The KD group also experienced significantly more problems associated with their conduct such as temper tantrums, disobedience and argumentative behaviour when compared with the hospital control group (P = 0.030). Greater total behavioural difficulties experienced within the KD group when compared to the hospital controls were also demonstrated (P = 0.048).

On the Parental Stress Index, the KD group achieved greater mean scores compared to the control groups. However, the differences fell short of significance. There may be a trend here towards a 'difficult' child being the main source of stress within the parent-child relationship (P = 0.084), but further research is required to confirm this.

No significant differences were shown between the hospital control and the sibling control groups within any of the questionnaires.

13 patients within the KD group were documented as suffering or having suffered from coronary artery dilatation or aneurysms secondary to their Kawasaki disease. No significant differences or trends to significance were shown when comparing the mean behavioural scores of the KD group with dilatations/aneurysms and the KD group without dilatations/aneurysms.

Furthermore, no significant differences or values approaching significance were demonstrated when comparing age of onset of Kawasaki disease with mean behavioural scores.

## Discussion

Our initial indications that a significant number of children experience behavioural sequelae in the short to medium-term post Kawasaki disease was confirmed by more detailed investigation. The KD group showed higher scores on measures of behavioural and emotional difficulties when compared with both control groups, with the emergence of significant differences demonstrated between the KD group and the controls across various categories of the questionnaires. The CBCL showed these significant differences to exist between the KD group and the sibling control group, whereas the SDQ showed significant differences between the KD and the hospital control group.

Like King et al. (2000) [7], we have shown a strong preponderance towards problems of an internalising nature within the KD group. 40% of the KD group were shown to have internalising scores in the clinical or borderline-clinical range, representing an incidence greater than twice that of the control groups. In line with this, significantly higher scores were attained by the KD group compared to sibling controls within the internalising sub-categories of somatic problems and withdrawn traits. However, unlike King et al., our results also highlight further difficulties relating to thought and conduct existing within the KD group.

Importantly for parents, no significant differences were demonstrated between the groups with regard to participation in activities and hobbies, social interaction or performance at school. This, hopefully could form the basis of some reassuring parental advice.

However, despite the lack of any proven link between adverse effects on the child's overall social and academic performance, the high incidence of clinical and borderline-clinical internalising behaviour should make the paediatrician aware of the potential need for referral to a clinical psychologist during long term management. On account of the fact that internalising behaviour can be harder to detect than overt externalising traits, some simple screening questions into the child's behaviour should be posed to the parents during long-term follow up of Kawasaki disease.

This study has a few potential sources of bias. The relatively low return rate of the questionnaires raises the possibility of self selection bias. Bias originating from the parent answers to the questionnaires may also exist. Kawasaki disease is a relative rare disease with the potential for serious cardiac complications. The associated fears and anxieties likely to be experienced by the parents may serve to exaggerate any observed behavioural difficulties in the minds of the parents. This would be reflected in higher than expected behavioural scores. However, the CBCL has been argued to mitigate this bias and moreover, Green et al (1998) suggest that the CBCL is in fact intrinsically conservative in discriminating mild emotional and behavioural problems [16]. The relatively small size of the groups, especially the controls, may have limited the extent of the significant behavioural and emotional problems seen, and we would therefore expect to see more significant results emerge should the size of the groups be expanded.

### Pathological associations

Kawasaki disease is a systemic vasculitis which seems to have a predilection to coronary arteries, although it has also been shown to affect the cerebral arteries. Evidence suggesting the nature of the consequences arising from the cerebral vasculopathy has come from a single-photon emission computed-tomography study carried out on 21 children in Japan by Ichiyama and Nishikawa. They were able to demonstrate localised cerebral hypoperfusion occurring in 29% of the patients during the acute phase of Kawasaki disease [17].

Systemic lupus erythematous is also a multi-organ systemic vasculitis, which is known to affect the cerebral nervous system (CNS), causing various neuropsychiatric symptoms [18,19]. Multiple areas of hypoperfusion have been shown on SPECT studies of lupus patients positive for neuropsychiatric symptoms [20]. Interestingly, one study of 35 patients by Colamussi et al. (1995) found that in the absence of neuropsychiatric symptoms, uptake was normal in nearly all of the patients, whereas hypoperfusion was demonstrated in most patients with severe neuropsychiatric sequelae [21].

It is suggested that these areas of hypoperfusion documented within lupus and Kawasaki disease may be caused by a process of cerebral vasculitis resulting in patchy ischaemic areas. The damage to the CNS which is likely to arise may then alter neurological function for some time after the acute phase of the illness, and this would certainly provide one reasonable explanation for the behavioural difficulties that have been shown to persist in the medium to long-term.

Indeed, supporting evidence comes from Suzuki et al, (2000) who suggested that the remodelling of coronary aneurysms is a process that continues for many years after the onset of the disease [22]. One might surmise that continued behavioural problems may be the result of a parallel remodelling process occurring in the brain.

Thus, the evidence of CNS pathology adds further weight to our belief that the behavioural changes arise secondary to a cerebral vasculopathy, and are not merely due to the psychological complications of an acute severe illness.

### Further experimental evidence

In order to further investigate any identifiable CNS pathology affecting the brain, active or otherwise, we have performed PET (glucose and ammonium) and MRI scans on 7 post Kawasaki disease patients with severe behavioural difficulties (Table 4). In all seven cases these were carried out at least 6 months after their last episode of Kawasaki disease. Three of these patients were reported to have slightly reduced uptake of tracer, occurring within the occipital lobes, although other areas were also affected in two of the cases. Although these minimal changes are of uncertain significance, they could represent resolving areas of cerebral hypoperfusion secondary to cerebral vasculitis that occurred during the acute phase of the disease. An ethically approved study with a larger patient base would be the next step to investigate these changes further.

**Table 4:** Cerebral PET scan results following acute Kawasaki disease

**No.**	**Year of Kawasaki disease**	**PET scan result**	**Date of PET scan**
1	1997	Diffusely reduced uptake in occipital lobes and cerebellum	24/6/99
2	1999	Normal	20/9/00
3	1999	Asymmetry in occipital region lower on R side	20/3/00
4	1995	Normal	27/5/99
5	1997	Normal	20/5/99
6	1999	Slight reduction in posterior parietal and occipital lobes symmetrically.	28/10/99
7	1997	Normal	7/7/99

### Summary

In conclusion, we have shown significant behavioural sequelae can occur following acute Kawasaki disease. Therefore, the paediatrician should bear in mind the potential need for long term follow up of Kawasaki disease patients in clinic even in the absence of coronary artery aneurysms. Parents can be told that behavioural difficulties experienced may be within the normal sequelae of the disease process and they can be reassured that they do not appear to affect school performance to any significant degree. However, a few probing questions should be asked to flesh out other possible causes before any difficulties are attributed to Kawasaki disease. The paediatrician should consider referral to a clinical psychologist where necessary. This may indeed prove to be a common requirement in long-term management, when it is considered that 40% of the KD group in this study fell within the clinical or borderline-clinical range for internalising problems.

Future research should examine the behavioural difficulties in greater detail, employing formal neuropsychiatric testing. Additionally, further research in this area is required to rule out other possible causes of behavioural sequelae. In particular, standard treatment with aspirin and intravenous gammaglobulin should be eliminated as potential aetiological factors.

## Pre-publication history

The pre-publication history for this paper can be accessed here:


